# Pharmacy-based methadone treatment in the US: views of pharmacists and opioid treatment program staff

**DOI:** 10.1186/s13011-023-00563-w

**Published:** 2023-09-11

**Authors:** Li-Tzy Wu, Paolo Mannelli, William S. John, Alyssa Anderson, Robert P. Schwartz

**Affiliations:** 1 Department of Psychiatry and Behavioral Sciences, Duke University School of Medicine, Durham, NC USA; 2 Department of Medicine, Division of General Internal Medicine, Duke University School of Medicine, Durham, NC USA; 3 Center for Child and Family Policy, Sanford School of Public Policy, Duke University, Durham, NC USA; 4 Duke Institute For Brain Sciences, Duke University, Durham, NC USA; 5 Cardinal Health, Dublin, OH USA; 6 IQVIA, Durham, NC USA; 7 Friends Research Institute, Inc, Baltimore, MD USA

**Keywords:** Community pharmacy, Methadone maintenance treatment, Methadone medication unit, Opioid use disorder, Opioid treatment program, Methadone prescribing

## Abstract

**Background:**

The US federal regulations allow pharmacy administration and dispensing of methadone for opioid use disorder (PADMOUD) to increase the capability of opioid treatment programs (OTPs) in providing methadone maintenance treatment (MMT) for opioid use disorder (OUD) as part of a medication unit. However, there is a lack of research data from both pharmacy and OTP staff to inform the implementation of PADMOUD.

**Methods:**

Staff of a pharmacy (n = 8) and an OTP (n = 9) that participated in the first completed US trial on PADMOUD through electronic prescribing for methadone (parent study) were recruited to participate in this qualitative interview study to explore implementation-related factors for PADMOUD. Each interview was recorded and transcribed verbatim. NVivo was used to help identify themes of qualitative interview data. The Promoting Action on Research Implementation in Health Services (PARIHS) framework was used to guide the coding and interpretation of data.

**Results:**

Six pharmacy staff and eight OTP staff (n = 14) completed the interview. Results based on PARIHS domains were summarized, including evidence, context, and facilitation domains. Participants perceived benefits of PADMOUD for patients, pharmacies, OTPs, and payers. PADMOUD was considered to increase access for stable patients, provide additional patient service opportunities and revenues for pharmacies/pharmacists, enhance the capability of OTPs to treat more new patients, and reduce patients’ cost when receiving medication at a pharmacy relative to an OTP. Both pharmacy and OTP staff were perceived to be supportive of the implementation of PADMOUD. Pharmacy staff/pharmacists were perceived to need proper training on addiction and methadone as well as a protocol of PADMOUD to conduct PADMOUD. Facilitators include having thought leaders to guide the operation, a certification program to ensure proper training of pharmacy staff/pharmacist, having updated pharmacy service software or technology to streamline the workflow of delivering PADMOUD and inventory management, and reimbursement for pharmacists.

**Conclusion:**

This study presents the first findings on perspectives of PADMOUD from both staff of a community pharmacy and an OTP in the US. Finding on barriers and facilitators are useful data to guide the development of strategies to implement PADMOUD to help address the US opioid crisis.

## Introduction

Since 1999, the overdose epidemic in the United States (US) has resulted in over a million deaths [1, 2]. In 2021, there were an estimated 106,699 drug overdose deaths, representing a nearly five-fold increase in the age-adjusted rate per 100,000 population between 2001 and 2021 [3]. Despite the ongoing opioid epidemic, most individuals who may benefit from treatment with Food and Drug Administration (FDA)-approved medications for opioid use disorder (MOUD) do not receive such treatment [4–6]. This treatment gap is in part due to shortages of opioid treatment programs (OTPs) and practitioners who provide treatment with MOUD (e.g., methadone, buprenorphine, and extended-release naltrexone) [7, 8].

There is an urgent need to increase access to OTPs that provide methadone maintenance treatment (MMT). MMT has been available in the US for over 55 years, is highly effective, and is the most studied MOUD [9, 10]. However, access to the nearly 2,000 OTPs in the US has been constrained by US federal regulations on methadone dispensing [11, 12]. In the first months of MMT admission and during periods of instability, patients must attend the program at least six days/week for direct dose administration. The US regulations permit stable patients who have been successful in treatment for at least one, two, or three years to receive up to 6, 14, and 30 take-home doses, respectively [13]. The limited number of OTPs, the high attendance burden early in treatment, and the concentration of most OTPs in metropolitan areas limit the number of patients who can access and remain in treatment [11, 14]. By comparison, there are nearly 68,000 pharmacies in the US that are distributed geographically more widely than OTPs [15].

Longer drive times and higher transportation costs in terms of time and public transportation costs are related to missed methadone doses, poor treatment retention, poor quality of life, or difficulties maintaining employment [16, 17]. For example, patients who lived 10 + miles from an OTP were more likely to miss methadone doses than those who lived within 5 miles of an OTP [18]. Thus, establishing methadone treatment closer to patients’ homes would likely improve patient outcomes. Pharmacies are an underused resource in MMT in the US that could support pharmacy administration and dispensing of methadone for opioid use disorder (PADMOUD), could increase access to treatment, reduce long drive and public transportation times to attend treatment, improve retention, and help patients maintain their employment. Outside of the US, pharmacies are used to provide MMT or address shortages in practitioners in other countries [19–21]. By implementing pharmacy dispensing of methadone, MMT in Canada is considered the first-line treatment for opioid use disorder (OUD) that has been able to enroll harder-to-reach patients (e.g., young adults) [22]. The rates of individuals receiving methadone in Canada are estimated to be 3–4 times higher than rates in the US [22].

There are two ways in which pharmacies could provide MMT. The first has been a part of the US federal regulations for decades and allows OTPs to collaborate with community pharmacies to establish a “Medication Unit” (MU) with approvals from proper federal (SAMHSA, Drug Enforcement Administration [DEA]) and state agencies [12]. The federal regulations define an MU as a facility established as part of, but geographically separate from, an OTP from which licensed practitioners or pharmacists administer and dispense methadone for OUD treatment [12]. A MU can be a facility/unit owned by the OTP or a pharmacy MU staffed by licensed pharmacists and under the oversight of the parent OTP through a practice service agreement between an OTP and a pharmacy. Establishing an MU is possible but has a number of administrative and logistic limitations (e.g., methadone storage and records must be kept separate by the pharmacy from methadone stored for analgesia, the medication must be shipped to the OTP which in turn must bring the medication to the pharmacy). The second way for a pharmacy to provide MMT would be for a physician to prescribe methadone for the treatment of OUD and the pharmacy [pharmacists] to administer and dispense the medication, which is not permitted under the current federal regulations [23].

There is a lack of research data on pharmacy MUs operating under current federal regulations to guide the implementation of pharmacy MUs and on pharmacy-delivered MMT through prescription. To fill this gap, under federal regulatory exemption, Wu and colleagues [23] conducted a clinical trial on PADMOUD via electronic prescribing of methadone from an OTP physician to an independent pharmacy (e.g., pharmacist-owned rather than a large corporate chain) to test feasibility of this innovative approach in the US. Wu et al. obtained federal exemption approvals to enroll 20 established and stable OTP patients to transfer their methadone administration and dispensing to an independent community pharmacy via electronic prescribing for three months [23]. The study found a high treatment retention rate, perfect indicators of treatment fidelity (i.e., pharmacist-delivered PADMOUD), no methadone-related safety events, and no illicit drug use based on urine drug screens [23]. To understand implementation-related factors for providing PADMOUD in the US, the present qualitative study investigates the perspectives of both OTP and community pharmacy staff that were the study sites of the clinical trial of PADMOUD [23].

## Methods

### Parent study design

The parent study design and its primary findings were reported elsewhere [23]. In brief, it was conducted within an OTP (one prescribing physician) and one independent community pharmacy (two pharmacists) located 5.4 miles from the OTP in North Carolina, US. Because the US regulations do not allow methadone to be prescribed for OUD treatment, the parent study obtained exemption approvals from the US Drug Enforcement Administration, SAMHSA, and NC State Opioid Treatment Authority and approval form the Duke University Health System’s Institutional Review Board to conduct the trial [23].

A collaborative practice agreement was established between the OTP and pharmacy to specify the pharmacist and physician roles and responsibilities for operating PADMOUD [23]. The physician was responsible for the treatment plan, prescribing methadone and dose adjustments, keeping records for federal/state regulations, and providing clinical guidance/coaching and supervision of the pharmacists, who in turn administered and dispensed methadone to the patient. Clinical activities performed by the pharmacists at each visit were recorded on a methadone visit checklist for evaluating the intervention’s fidelity. These activities included methadone reconciliation, safety assessments, checking the patient’s prescription drug monitoring program report prior to dispending methadone, providing patient education or counseling, communicating with the OTP physician regarding any concern, administering one methadone dose at the pharmacy and dispensing methadone according to the prescription. The tasks of PADMOUD was summarized in the parent study [23].

Research staff screened and enrolled patients eligible to receive between 6 and 13 days of take-home methadone doses from the OTP. The OTP physician prescribed methadone electronically for participants to have their methadone administration and dispensing of take-home doses transferred to the pharmacy for 3 months. Methadone was provided in tablet formulation matched to their dosage from the OTP for the study using 40-mg dispersible tablets for oral suspension and/or 5-mg non-dispersible tablets. Participants picked up their methadone take-home doses from the pharmacy regularly based on their allowed take-home schedule from their OTP treatment plan. Prior to dispensing take-home doses at each pharmacy visit, the pharmacist observed ingestion of one dose at the pharmacy. During the study period, participants continued to receive drug testing and counseling as usual at the OTP. At the end of the study, participants returned to the OTP for routine methadone administration/dispensing. Qualitative interviews were conducted after the completion of the parent study.

### Qualitative study design

#### Participants

A purposive sampling method was used to recruit staff of the pharmacy and the OTP that were study sites of the parent study [23]. One female interviewer with training in psychology (AA) who conducted the study’s patient participant assessments for the parent study recruited the pharmacy and OTP staff for the qualitative interviews by email or phone call between January and March 2021. Their self-reported demographic information is found in Table 1. Of 8 pharmacy staff available for recruitment, 6 staff completed the interview (1 declined; 1 did not respond) including four pharmacists and two pharmacy staff. Of the 9 OTP staff available for recruitment, 8 staff completed the interview (1 did not respond) including the medical director, one program director, two nurses, two counselors, and two OTP staff. Each participant provided informed consent and received $50 for compensation of time.

**Table 1:** Participant characteristics (n = 14)

Characteristic	n (%)
**Role at the facility**	
Pharmacist	3 (21.4)
Other pharmacy staff	3 (21.4)
Opioid treatment program physician	1 (7.1)
Opioid treatment program director	1 (7.1)
Opioid treatment program staff (e.g., nurse, counselor, other staff)	6 (42.9)
**Sex**	
Male	6 (42.9)
Female	8 (57.1)
Age in years*	
18–35	6 (42.9)
36–70	7 (50.1)
**Ethnicity**	
Not Hispanic or Latino	14 (100)
Hispanic or Latino	0
**Race**	
White	10 (71.4)
Black/African American	3 (21.4)
Other	1 (7.1)
**Education completed**	
High school graduate/GED or less	0
Some college or more	14 (100)
**Professional degree (based on the education question)**	
Some college, no degree	2 (14.3)
Associate	2 (14.3)
Bachelor	2 (14.3)
Master	4 (28.6)
PharmD	2 (14.3)
MD or DO	2 (14.3)

* Missing data on age (n = 1)

#### Data collection

Given the lack of research data on PADMOUD and its implementation, we used the Promoting Action on Research Implementation in Health Services (PARIHS) framework and relevant studies of PADMOUD to design interview questions [24–28]. The PARIHS framework identifies domains (e.g., factors) related to the evidence (e.g., perspectives on intervention/PADMOUD), context (e.g., pharmacy/OTP capability and intention), and facilitation (strategies for addressing barriers and promoting facilitators) to inform future implementation of PADMOUD. Hence, interview questions asked about participants’ perspectives on PADMOUD (e.g., benefits/disadvantages), context-related factors (capability and intention to support), and facilitation-related factors (barriers, facilitators, and recommendations) [24–28]. Example interview questions are displayed in Table 2. The interview study took approximately 40-60 min. All interviews were conducted by zoom. Each interview was audio-recorded and transcribed verbatim by the interviewer (AA). Transcripts were not returned to participants for comments.

**Table 2:** Example interview questions and domains related to the Promoting Action in Research Implementation in Health Service (PARIHS) framework

PARIHS domain	Construct	Interview questions
Evidence: perceptions of the Intervention	Perceived benefits and disadvantages (pharmacy vs. OTP)	♣ Do you think pharmacist-provided services for methadone treatment (e.g., dosing and dispensing take-home doses) could be important for individuals with opioid use disorder? ♣ Can you please describe what you think are the potential advantages and benefits of methadone dosing and dispensing take-home methadone doses in a community pharmacy setting? What about potential disadvantages or negative outcomes?
Context: readiness of the context	Ability to perform	♣ How comfortable do you think pharmacists and pharmacy staff, in general, would be with dosing and dispensing methadone for opioid use disorder? ♣ How effective do you think pharmacies could be at dosing and dispensing methadone? ♣ How comfortable do you think pharmacists and pharmacy staff, in general, are with discussing patients’ substance use and treatment? ♣ How comfortable do you think clinics would be transferring their clients’ methadone dosing and dispensing to a local community pharmacy?
Context: readiness of the context	Intention to support PADMOUD	♣ Do you think pharmacists and pharmacy staff in general would be supportive of methadone dosing and dispensing for opioid use disorder at a pharmacy? ♣ Do you think methadone clinic staff (e.g., doctors, counselors, and nurses) in general would be supportive of methadone dosing and dispensing for opioid use disorder at a pharmacy? ♣ How do you think health insurance payers would feel about methadone dosing and dispensing methadone for opioid disorder at a community pharmacy? ♣ Who or what would most influence your decision to provide or support methadone dosing at a pharmacy?
Facilitation: strategies to address barriers and promote implementation	Barriers, facilitators, and strategies	♣ What factors or circumstances do you think would make it difficult or impossible for patients to receive take-home methadone doses from a pharmacy? ♣ What do you think would be the main barriers for opioid treatment program providers and clinics to implementing pharmacy-based methadone dosing and dispensing? ♣ What do you think would be the main barriers for pharmacists and pharmacies to implementing pharmacy-based methadone dosing and dispensing? ♣ What type of support do you think they would need to most effectively implement methadone dosing and dispensing at the pharmacy? ♣ What would be some good strategies for fitting methadone dosing and dispensing into the clinic’s regular workflow?

PARIHS: Promoting Action in Research Implementation in Health Services (PARIHS) framework

### Data analysis

We used a flexible coding approach to guide the coding procedures and identification of themes [29]. After data collection, two investigators (LTW and WSJ) reviewed all transcripts for completeness and developed an initial set of index codes based on the interview guide and relevant research findings on PADMOUD [23–28] and inductively derived from themes emerged in the initial review of transcripts. We used initial reviews of transcripts to become familiar with the data. One investigator (WSJ) then applied these index codes to all interview data and identified excerpts for index codes using the NVivo software [30]. One investigator (WSJ) summarized findings of index codes and related themes/sub-codes emerged from the analysis and tabulated identified codes/themes by subject ID. Two investigators (PM and LTW) conducted independent reviews of all transcripts and coding and summarized identified codes/themes. Investigators (WSJ, LTW, and PM) then discussed discrepancies in coding to resolve differences. In summary, we used the NVivo software to organize data and find insights in data, and additional reviews and coding by two investigators to enhance the reliability and validity of finding themes/codes commonly emerged from the data.

## Results

### Demographics

Of the 14 participants, 57.1% (n = 8) were female, 50.0% (n = 7) were aged 36–70 years, and 71.4% (n = 10) were white (Table 1).

### Qualitative interview findings

Results emerged from the data were consistent with three domains of the PARIHS framework. These findings are organized based on these domains: evidence, context, and facilitation.

### Evidence (perspectives on PADMOUD)

The evidence domain concerns participants’ perception of the intervention (benefits and disadvantages of PADMOUD). Participants reported benefits/advantages of PADMOUD for patients, OTPs, pharmacies, and payers.

#### For patients

PADMOUD was considered a treatment option that could increase access for patients by offering a more convenient setting (pharmacy) and flexible office hours, less waiting time to receive medication, and fewer drug use cues/triggers (e.g., less contact with drug users) to prevent relapse than usual care at the OTP.

*Pharmacy participant # 1: “The advantages are access, improved access, improved schedule, flexibly, and decreased triggers.”*

*OTP participant # 1: “If it’s a 24-hour pharmacy and you’ve got the flexibility of picking up your week’s prescription, whenever you want on the day that it’s due, that creates a lot more flexibility.”*

In particular, time was considered a key factor underlying the support for PADMOUD:

*Pharmacy participant # 3: “The time factor is probably the most frustrating part of not only their access to treatment and ability to stay adherent and things like that. Everyone struggles with taking more time to do things that all frustrate us. So I think that’s [PADMOUD] a huge thing that make it easier for patients.”*

PADMOUD was considered a good option for stable patients, as it would allow patients to step down from a more-structured setting to a less-structured setting where patients could go for their medication at flexible hours, live more normal lives, or maintain regular work schedules:

*OTP participant # 6: “For stable patients, it [PADMOUD] possibly would make it easier because they’re not coming into the clinic, and they’re not having to wait in line. Many of them have jobs to get to, so they’re rushing.*

*OTP participant # 7: This [PADMOUD] would definitely be a draw for them to seek this service. So this is kind of a step down from the higher structure to a lower structure where they can go and not have to worry about the hours. And they just don’t need the counseling twice a month, quite as much as they don’t need nurses to see them in person.”*

However, PADMOUD was considered less suitable for non-stable patients with clinical issues that require frequent urine drug screens and/or counseling:

*Pharmacy participant # 2*: *“If a patient was getting more or having more frequent urine drug screens or that kind of thing in a clinical setting, that [PADMOUD] might not be an option in a pharmacy setting, but that could obviously be part of the collaboration between decisions about the physician, what the patient needs.”*

In addition, patients would go to multiple locations for their treatment, which would increase the challenge monitoring patients’ compliance with methadone treatment:

*OTP participant # 2: “The barriers would definitely be their drug screens and their compliance. I know that they’re going back to the main clinic for drug screening and counseling. So a disadvantage would just be like having to go to multiple locations for their services.”*

#### For OTP/OTP staff

PADMOUD was considered an option to increase OTPs’ capability to treat more patients and reduce OTP staff burden.

*OTP participant # 6: “For the methadone clinic, this would help reduce the traffic that comes through for the staff, especially on the weekends. It’s a lot of responsibility to dose all those patients in that short amount of time.”*

Although PADMOUD could decrease the OTP’s revenues, PADMOUD was perceived to give the OTP an opportunity to treat more new patients to increase revenues:

*Pharmacy participant # 1*: *“Methadone clinics themselves could decrease their volume and decrease their overall revenue, but it could give them potential opportunities to work with other community providers to decrease or increase their overall volume, which could treat more patients and give them opportunity to continue their revenue, at the same rate.”*

Further, PADMOUD was considered to increase the OTP’s burden of communication and record keeping:

*Pharmacy participant # 3: “Con [disadvantage of PADMOUD] is associated with increased record keeping in terms of communicating changes and dosing with the pharmacy and making sure they get implemented on a real time manner.”*

Therefore, streamlining the workflow processes among OTP staff, patients, and pharmacy staff was considered important to ensure proper operations of PADMOUD (e.g., coordination and communication on record keeping, medication orders/supply, methadone doses, drug screen, and psychosocial counseling):

*OTP participant # 5: “What days do the clients have drug screen? It’s very hard to get the drug screens like all of those things that are still necessary, but making sure that it’s streamlined a little bit better where there’s a lot more structure.”*

#### For pharmacy/pharmacists

PADMOUD was considered to increase pharmacists’ skills and promote collaborative opportunities with physicians.

*Pharmacy participant # 4: “It [PADMOUD] adds a new skill. I guess it’s also kind of cool to see that you’re helping someone instead of just filling a prescription or checking a prescription and sending it off. You are actually getting that one-on-one patient interaction.”*

*Pharmacy participant # 2: “I think advantage again, just increasing more collaborative work between pharmacists and physician practices. Just the opportunity to have that kind of role in patient care is a is a big advance for pharmacists.”*

Additionally, PADMOUD would increase revenues for pharmacies/pharmacists:

*Pharmacy participant # 1*: *“Most pharmacists are very hungry for medical opportunities and ways to get reimbursed. Like why not also pick up your maintenance medications? So getting that [PADMOUD] also increases the profit for the pharmacies, increases the sync status and the accessibility to the other medications that they’re taking.”*

Although participants perceived a potential concern of liability (e.g., issues related to patient relapse or overdose) among pharmacists, liability could be addressed by receiving additional training on assessing and monitoring signs of relapse or overdose problems:

*OTP participant # 5: “I think it is a big liability because like I said, relapse. And they [pharmacists] not having a baseline of what the client looks like and not being able to kind of tell if this client is not okay, because they’re going to be the eyes and ears for the clinic between times when they’re not doing their drug screens. So being able to 1) give those pharmacists education and 2) being able to help the pharmacist kind of watch out for some of the warning signs.”*

Another issue for pharmacy/pharmacists was the time burden for delivering PADMOUD and identification of pharmacies willing to take on the additional workload and responsibility:

*Pharmacy participant # 6: “It just takes more of a pharmacist’s time for them to see the patient and do methadone dosing and just interacting with them would take more time for the pharmacist.”*

*Pharmacy participant # 3: “I think maybe finding pharmacies that are willing to take on the additional workload and responsibility. I think that’s definitely the biggest barrier to finding the pharmacies that want to take on the additional workload.”*

#### For payers

PADMOUD was considered acceptable to payers (e.g., insurance companies), as it would have lower costs for patients than going to the OTP (e.g., dispensing costs).

*OTP participant # 1: “I guess the advantage for the health insurance payers is typically we get paid, you know, like 16 dollars a dose, but at a pharmacy, the usual reimbursement is like five dollars to fill a prescription. So it may be cheaper for them to use a pharmacist as opposed to an OTP.”*

*Pharmacy participant # 2: “It would be less expensive for insurance companies. There would be some cost benefit from both the provider fees as well as the medication fees.”*

### Context (capability and intention)

The context domain was related to the perceived capability of pharmacy and OTP staff to implement PADMOUD and the perceived support from pharmacy/OTP staff and payers (e.g., insurance company).

#### Pharmacies’ ability to implement

Participants perceived that pharmacists would be comfortable and effective in delivering PADMOUD if pharmacists received the right training and had the PADMOUD protocol in place.

*OTP participant # 2: “I think they [pharmacists] could be very comfortable at dispensing. It’s all about the team of people you’re working with, communication, as far as training that is available for them and the tools, you know, binders, whatever they need as far as those resources. So if they have a good team, the doctors and everything where they can go back and if they have questions, ask the questions, things like that, I think that will be fine.”*

*Pharmacist participant # 2: “Pharmacies can be very effective at dosing, dispensing methadone, but it would just require the right types of tools and equipment for that to happen.”*

Regarding whether pharmacists and pharmacy staff, in general, would be comfortable with discussing patients’ substance use and treatment, participants also considered that pharmacists would need additional training and resources to help with their skills:

*OTP participant # 3: “My experience with pharmacists have been that they don’t know what to do with the people who have the issue [OUD], but they are very willing to share education. I think I’ve seen that more in probably the last 10 years. I’ve just been more aware of the education component with pharmacists. In both small and large pharmacies now, the first thing out of their mouth is, do you have any questions? So I think that they may not know what to do with it, but I think that they have education, they have some stuff they might be able to give somebody to read. So I think they are a little more comfortable with discussing substance use treatment.”*

In particular, building trust in relationships with patients would improve pharmacy staff’s skills in discussing patients’ substance use and treatment:

*Pharmacy participant # 6: “I guess it would be easier for pharmacy staff to discuss this with the patients as long as patients have more trust in the pharmacist. I guess we are not uncomfortable at all with discussing substance use with the patients if we are building trust and having its familial trust with the patients, ongoing patients, not just new patients.”*

#### OTPs’ ability to implement

OTP programs would be comfortable transferring their patients’ methadone dosing and dispensing to a local community pharmacy when patients were relatively stable and communication procedures between the OTP and the pharmacy were in place.

*OTP participant # 6: “What would make them comfortable if it’s the right patient, patients that have definitely demonstrated progression in the program with their opiate use treatment, and the right communication between the pharmacy and the clinic.”*

Participants also perceived that pharmacy staff should have received training on PADMOUD and have a proper protocol in place to ensure a smooth transition of patient treatment from an OTP to a pharmacy:

*OTP participant # 8: “What would make them feel more comfortable is going to be a nice transition where everyone can kind of understand each other and everything’s kind of passed over smoothly. And all the information is transferred over and we feel like they’re in good hands. And the people who are taking care of them are educated on what the treatment is.”*

#### Perceived support from OTPs

Participants perceived positive support from OTP staff because PADMOUD was considered a good option for stable patients (e.g., a step down).

*OTP participant # 3: “The physicians, the counselors and the nurses would support additional options, treatment for our clients. So a step down is something I think that they would see. I definitely think that it should be for patients that are stable.”*

Another reason for support was reduced staffing time with stable patients to allow the OTP to treat additional patients (e.g., new or severe patients):

*Pharmacy participant # 6: “From staff perspective, I guess it is like less work for them to do this if pharmacy is being used for methadone dispensing.*

*Pharmacy participant # 4: “It can alleviate some of the in and out of their [OTP] office and the time that it takes away to diagnose other patients and to help their other patients.”*

A further reason was to respect patient preference for going to a pharmacy, as it was considered to improve patients’ treatment compliance:

*OTP participant # 2: “If the client wants to be there, we have to support what makes them comfortable and compliant with their dosing. The reason was because we were able to see our clients who were successful, the pharmacy based treatment, and being able to hear our clients talk about how much of a relief it was.”*

However, some for-profit OTPs might not support PADMOUD due to reduced revenues:

*OTP participant # 4: “For clinics that are for profit, that’s going to be an issue. If you’re going to lose money by sending them out, then that’s not going to work.”*

Nonetheless, other for-profit OTPs might still support PADMOUD for the reason of helping patients (e.g., improved treatment access):

*OTP participant # 8: “The idea is that we can help more people, the better, even if it’s less money in our pocket. As long as we can get people the help that they need and the treatment they need, the right way, we can support. That’s how we function here.”*

#### Perceived support from pharmacies

Participants also perceived positive support from the pharmacy staff because it would meet the needs of the underserved populations and provide additional revenue.

*Pharmacy participant # 1: “The majority of pharmacy staff would be very supportive of bringing an alternative revenue to help solidify longevity to provide long term care security for those individuals and also give them opportunities to reach a desperately needed patient population.”*

*Pharmacy participant # 4: “For the advantage of helping people get off their addiction. It would help the community. Just knowing that they have a place that they can go to get the help that they need.”*

Independent pharmacies were perceived to be more suitable than large chain pharmacies in implementing PADMOUD due to extra time required to interact with patients receiving methadone:

*Pharmacy participant # 3: “I think more so with the independent pharmacies. They would be a better way to do it or even an independent chain. More so because they have the additional time to interact with that population that it requires, because basically every interaction is almost the same amount of time as like a flu shot; whereas the chains, I think that would be just more of another hurdle for them.”*

The Community Pharmacy Enhanced Services Network (CPESN) was considered a suitable network of pharmacies to implement PADMOUD, because pharmacists of the CPESN would have more time available to interact with patients:

*Pharmacy participant # 3: “I have heard from different kinds of chains like CVS and Walgreens. I feel like it would be a little bit more difficult to maybe implement that reduced stigma on a companywide level. So maybe like through the Community Pharmacy Enhanced Services Network (CPESN). I think that may be a better route of going with those kinds of pharmacies who also have a little bit more time on their hands to actually forge that interaction and that kind of ties in the pharmacies.*

#### Perceived support from payers

Participants also considered support from insurance payers because PADMOUD would save costs.

*OTP participant # 1: “Insurance payers would be fine with it, especially if it would save them money. I think that’s what their focus is going to be. So I would think that they would be supportive of that.”*

### Facilitation (strategies to address barriers)

Participants provided important recommendations for facilitating the implementation of PADMOUD, including having thought leaders, collaborative agreements, education, pharmacy staff attitudes, pharmacy staffing, technology/workflow, inventory management, and reimbursement.

#### Thought leaders

Having thought leaders serving as resource would facilitate the setup and operations of PADMOUD.

*Pharmacy participant # 2*: “*Have people, sort of thought leaders, in that could serve as resources or references for groups that are monitoring pharmacies or clinics or physicians who are interested in getting this type of collaboration set up.”*

#### Collaborative agreements

It was considered critical to establish collaborative relationships between the OTP and the pharmacy for PADMOUD and to start with just one pharmacy in order to understand implementation issues before involving with multiple pharmacies.

*Pharmacy participant # 3: “Making sure that there is collaboration that exists and maybe starting it off small before it’s completely rolled out with a pharmacy to test out.”*

#### Education

Pharmacist/pharmacy staff training or education on opioid addiction and methadone treatment was perceived to be a primary facilitator to implement PADMOUD.

*Pharmacy participant # 2*: *“The type of support that would be needed to most effectively implement would be definitely education. It would definitely be sort of a different practice that many pharmacies are currently used to. I think feeling like you’ve got education, that you’ve got support, contacts within the physician group to reach out to if there were any questions or something right in the moment.”*

Proper training was perceived to be necessary to ensure that pharmacy staff would know how to provide information about addiction/methadone and assess for signs/red flags of drug use issues:

*OTP participant # 5: “Training. It’s very necessary for them not to just do something because they were told to, but understanding how methadone impacts the receptors, how methadone treatment works, how it lasts, because if a client asks you those questions, it would be beneficial for them to be able to answer it if asked.*

Participants recommended to have a certification program requiring a minimal number of educational hours on addiction and methadone, and to have final support available for supporting such education:

*Pharmacy participant # 1*: *“Perhaps some sort of certification program or minimal number of educational hours manually like CE credits per year to participate.”*

*OTP participant # 6: “Financial support will probably need to help with the training, help with the staff and help with the overall; maybe remodeling of the pharmacy area to accommodate this.”*

#### Pharmacy staff attitudes towards methadone (stigma)

Stigma towards methadone/addiction among pharmacy staff was considered an important barrier. Participants recommended additional education on PADMOUD for pharmacy staff, including benefits of PADMOUD as well as stigmas issues around methadone.

*OTP participant # 3: “There’s a stigma around methadone and the kind of people that are in methadone treatment. It is very important for us to take the stigma off methadone. Being able to transition to pharmacy based, will hopefully alleviate some of that stigma that’s associated with it in the community.”*

*OTP participant # 8: “As long as people were properly educated on it and the benefits and it is helpful in the difference between methadone and Subutex and the other options out there. I think that would that would make a huge difference.”*

Specifically, pharmacists/pharmacy staff were expected to show nonbiased attitudes towards patients:

*Pharmacy participant # 6:* “*So just treating patients like normal people, not judging people, not treating them with judgment on their previous actions.”*

*OTP participant # 2: “Just continuing with good attitudes and letting them know if they have any questions or they feel uncomfortable because some clients may feel not comfortable and just keep things in because they don’t feel like they are being accepted at the pharmacy.”*

#### Pharmacy staffing

Additional staffing was considered needed to have designated pharmacy staff for PADMOUD.

*Pharmacy participant # 1*: *“The barriers would definitely be pharmacy staffing. Having one or two key individuals that are responsible for working with those that subset of patients. Having at least two people that are kind of a primary point to work with a group.”*

#### Technology/workflow

Updating pharmacy’s computer system and allowing pharmacy staff access to electronic medical records of patients were considered important for facilitating communication and operations of PADMOUD (e.g., monitoring patients’ compliance with drug screens).

*OTP participant # 6: “Update the computer technology.”*

*OTP participant # 4: “If the pharmacy had the same electronic medical record that we did, they could just go into Methasoft and they could put in real time the data that we could see in real time about what happened. Real time communication about all of it, which is not insurmountable in today’s technology.”*

In addition, PADMOUD procedures would be incorporated into an existing pharmacy management software (e.g., PioneerRX) to streamline the pharmacy workflow:

*Pharmacy participant # 3: “Tools. It would pretty easily be implemented into the pharmacy workflow, at least within PioneerRX as the pharmacy management software.”*

#### Inventory management

Participants also recommended that pharmacies should have the right kind of space and security measures to ensure proper inventory storage and management.

*Pharmacy participant # 2*: *“Definitely the right storage situation to be able to keep the medication separate and secure.”*

#### Reimbursement

Finally, participants recognized that the pharmacy would need to provide PADMOUD to enough patients and receive proper reimbursement (e.g., dispensing fees) in order to have adequate financial support.

*Pharmacy participant # 3: “It’s essential that you schedule enough patients and make sure that the reimbursement is at the appropriate level for the dispensing fee so that it makes the full time equivalent of a pharmacist worthwhile for the pharmacy to provide this program.”*

## Discussion

The long-lasting opioid-involved overdose death epidemic and shortages of MOUD capability in the US indicate a clear need to implement pharmacy-based treatments for opioid use disorder (PADMOUD) to improve access to, and retention in, methadone treatment [8, 31]. PADMOUD in the US has been underutilized and understudied. The present study reports the first US findings on pharmacy and OTP staff’s perspectives on benefits of, and barriers to, implementing PADMOUD. Participants considered PADMOUD to benefit patients (e.g., convenience, increased treatment access, reduced drug use triggers, facilitating recovery for stable patients), OTPs (e.g., reduced staff workload, increased capability to treat newer patients), pharmacies (e.g., training opportunities for patient care, new revenues), and payers (lower costs for receiving methadone from pharmacies than the OTP). Participants perceived support from pharmacies, OTPs, and payers. They also identified strategies to mitigate barriers to implementation.

### Evidence (perspectives on PADMOUD)

Several non-US countries have utilized PADMOUD to increase access to methadone and reduce OUD-related morbidity [19–22, 32, 33]. In Australia, MMT has been provided through community pharmacies since 1985, and pharmacies are the most common dosing sites [19, 34]. In the UK, the proportion of pharmacies dispensing methadone/buprenorphine for OUD increased from 51% to 1995 to 63% in 2005 [21]. Methadone patients in the Canada typically receive their initial treatment at addiction treatment clinics/programs; after stabilization, patients then go to approved locations, including local pharmacies and physician’s offices, to receive their observed daily dosing and take-home doses [32, 35, 36]. In the UK and Australia, methadone patients stabilized clinically after initial treatment by their physicians also can go to pharmacies to receive their methadone doses [27, 35]. The US federal regulations allow PADMOUD, but pharmacies/pharmacists operating the PADMOUD must obtain fairly burdensome federal and state regulatory approvals as an OTP medication unit approval [12]. The experience of the parent study indicates that a collaborative practice agreement can be used as a formal strategy to specify responsibilities of both staff of the partnered pharmacy and OTP for successfully operating PADMOUD by prescription outside the constraints of a medication unit in the US [23, 37, 38].

The implementation of PADMOUD in non-US countries not only allows stable patients to see their prescribing physicians only few times monthly for physician visits, but also facilitates individuals residing in remote/rural areas to access methadone at pharmacies near their homes [32, 36]. As shown in these non-US studies [19–22, 32, 33], PADMOUD could lessen key barriers to attending OTPs frequently experienced by US patients, including a long driving time from home to an OTP (transportation time/cost), lengthy waiting time at the OTP to receive methadone, and inadequate numbers of OTPs in nonmetropolitan and areas [11, 16–18]. Studies in the UK found that “having local access to methadone to save their travelling time” and “long opening hours of pharmacies to enable flexibility in managing time” were critical factors for going to pharmacies for methadone [26, 39].

### Context

The results indicate that participants are supportive of PADMOUD for reasons of offering a good option for stable patients (e.g., transition for recovery), respecting patient preference, increasing treatment access for under-served patients, enhancing the OTP’s capability to treat new or severe patients, or producing revenues for pharmacies. Participants also perceived that payers (e.g., insurance companies) would support PADMOUD because of lower costs at the pharmacy than at the OTP (e.g., lower pharmacy dispensing fees). To our best knowledge, there are no US research data on pharmacy and OTP staff ‘s willingness to support PADMOUD. Nonetheless, data from studies conducted in the Australia and UK indicated that community pharmacists were willing to continue their involvement with PADMOUD or take on additional patients after they have involved with PADMOUD [39, 40]. Pharmacists in rural areas were particularly willing to deliver PADMOUD services [40]. Data from the Canada showed that patients living in rural/remote areas with limited access to treatment were particularly likely to retain in methadone treatment once PADMOUD was made available [36]. Thus, having the experience of involvement with PADMOUD could potentially promote positive views on PADMOUD for both patients and pharmacists [41].

Participants also recognized the need for pharmacists to spend additional time interacting with patients and identified pharmacies (e.g., independent pharmacies) affiliated with the Community Pharmacy Enhanced Services Networks (CPESN®) USA as particularly suitable venues for implementing PADMOUD [42]. CPESN® USA is a nationwide clinically integrated network structured to advance pharmacy practice, which currently includes over 3,500 community pharmacies participating in 49 local networks in 44 states [42]. CPESN® USA not only is a federal vaccine partner helping independent pharmacies prepare to deliver the COVID 19 vaccinations to their communities, but also has educated and trained pharmacists in each network to have the skill set to approach health plans and other medical side payer to share the CPESN model in improving outcomes and surpassing performance metrics [43]. Hence, PADMOUD could offer CPESN-affiliated pharmacists opportunities to develop additional skills, provide new pharmacy services through collaborating with OTPs, and increase revenues from delivering PADMOUD. Engaging CPESN-affiliated pharmacists into practicing PADMOUD could potentially expand access to methadone treatment nationwide, including nonmetropolitan/rural areas.

### Facilitators

PADMOUD was considered a new pharmacy service requiring additional training for pharmacists and pharmacy staff. Thought leaders were recommended to provide resources and guidance for implementing PADMOUD. Thought leaders could include partnerships between pharmacy mangers and OTP directors, and their roles and responsibilities could be specified by a collaborative practice agreement to formally establish collaborative relationships for the pharmacy MU to operate PADMOUD [23, 37].

Data from the UK pharmacists involved with PADMOUD found that pharmacists expressed the desire to receive additional training on multiple resources/strategies, such as engagement with stakeholders (e.g., methadone prescribers, local addiction teams and experts, social services, support groups, drug misusers), blood-borne diseases and prevention, methadone treatment (e.g., interaction, revision, withholding, long-term maintenance, and tapering) off), drug addiction (e.g., common problems, best practice, ongoing updates), counseling, signs of intoxication and drug use, new drugs and local trends, naloxone training, needle exchange, local workshops, support available for service users, and suboxone [28, 44]. Pharmacists’ preferred methods for drug misuse training were distance learning, local workshops, online materials, and webinars [28].

The Providers Clinical Support System (PCSS) is a national training and clinical mentoring project developed to address the US opioid overdose epidemic, and it trains health professionals to provide evidence-based treatments to patients with opioid use disorder [45]. As shown in two US clinical trials on pharmacist-provided services for MOUD (buprenorphine, methadone) [23, 38], the virtual and remote training platform of the PCSS can be used to provide resources and training for pharmacists, pharmacy staff, physicians, and clinical staff who plan to involve with PADMOUD. Requiring educational certification for pharmacists via each state pharmacy board, including a pharmacist’s guide to methadone for OUD, should help ensure patient safety [46, 47]. For example, the Canadian model specifies completion of mandatory initial training requirements for pharmacies planned to dispense methadone, which includes a designated manager and at least one staff pharmacist who will dispense methadone [47]. The Canadian model also requires completion of training update (i.e., every 5 years), and all training for pharmacists are free through funding from the Ministry of Health and Long-Term Care [47]. The Canadian model could be considered by the US policy-makers and relevant stakeholders.

Pharmacy staff’s attitudes towards patients also influence patients’ decision for going to pharmacies for methadone [26]. Training materials for pharmacy staff/pharmacists should also address stigma or negative attitudes toward methadone among pharmacy staff because it can potentially promote positive working relationships between pharmacy staff and patients and facilitate the development of mutual trust and respect [39]. The latter is a critical factor to ease patients’ concern of a lack of privacy and/or fear for experiencing discrimination at pharmacies [27].

Further, pharmacists implementing PADMOUD should designate a consultant room or a quiet and private area for PADMOUD to protect patients’ privacy concern [26]. Allowing pharmacists to have access to patients’ electronic medical records would facilitate real-time communication between pharmacy staff and OTP physicians and enhance efficiency of PADMOUD and patient safety [48]. Finally, pharmacy managers could leverage the existing pharmacy service software (e.g., PioneerRX) to update the software for streamlining the workflow of PADMOUD, including inventory management and billing management.

### Limitations

This qualitative study was conducted to identify both pharmacy staff’s and OTP staff’s perspectives on PADMOUD because such data are unavailable from US studies [49]. This study was not designed to produce findings with a high level of generalizability. These results were based on employees of a community pharmacy and an OTP that were study sites of PADMOUD for a duration of 3 months [23]. Data from other countries suggest that involvement with PADMOUD not only could improve personal knowledge of PADMOUD, but also could develop positive views on helping patients via PADMOUD [39, 40, 44]. There has been no previous US research data on both pharmacy and OTP staff’s perspectives of PADMOUD based on operation of PADMOUD in the real-world setting. The experience of being a study site of PADMOUD (i.e., parent study) allowed pharmacy/OTP staff to observe and/or experience PADMOUD for three months, which offered them a unique opportunity to provide meaningful recommendations for implementing PADMOUD in the US. Because of their recent involvement with PADMOUD in the real-world setting, the identified facilitators and barriers were compelling and useful for informing future operations and implementation of PADMOUD in the US to address the limited access to MMT. We are not aware of particular pharmacy chains’ policies on providing MMT. Therefore, results from this study are timely and could shape the development of US pharmacies’ policies or guidance on MMT.

## Conclusions

Leveraging ubiquity of community pharmacies in the US to implement PADMOUD could meaningfully increase the number of methadone dispensing locations without the burden of establishing new OTPs. It could be accomplished through the use of MUs under the current federal regulations or with regulatory change through the use of methadone prescription by OTP physicians filled through affiliated pharmacies. Participants considered PADMOUD to benefit patients (especially stable patients, those living in rural areas, and those with transportation barriers), OTPs (increased capability to treat/enroll more patients, pharmacies (additional patient care services and revenues), and payers (lower dispensing fees/costs when receiving methadone at a pharmacy). The use of collaborative practice agreements offers a feasible means for the OTP and the pharmacy to establish collaborative relationships and specify a workable PADMOUD protocol for delivering services [23, 37, 38]. Individual US state officials could help support federal regulations for PADMOUD by publishing regulations specifying procedures under which PADMOUD would operate in the state to promote its implementation [50] or simply by permitting pharmacies to operate under any new federal regulations without further state constraint. Finally, the US Centers for Medicare & Medicaid Services and insurance companies should support PADMOUD and specify reimbursement rates for pharmacies and OTPs that deliver PADMOUD services.

## Data Availability

The datasets used and/or analyzed during the current study are not publicly available due to the nature of the study design, but are available from the corresponding author on reasonable request.

## Abbreviations

CPESN: Community Pharmacy Enhanced Services Network
DEA: Drug Enforcement Administration
FDA: Food and Drug Administration
MMT: Methadone Maintenance Treatment
MOUD: Medications for Opioid Use Disorder
MU: Medication Unit
OTPs: Opioid Treatment Programs
PADMOUD: Pharmacy Administration and Dispensing of Methadone for Opioid Use Disorder
SAMHSA: Substance Abuse and Mental Health Services Administration
OUD: Opioid Use Disorder
US: United States
